# Salvianolate injection in the treatment of unstable angina pectoris: A systematic review and meta-analysis

**DOI:** 10.1097/MD.0000000000005692

**Published:** 2016-12-23

**Authors:** Dan Zhang, Jiarui Wu, Shi Liu, Xiaomeng Zhang, Bing Zhang

**Affiliations:** Department of Clinical Pharmacology of Traditional Chinese Medicine, School of Chinese Materia Medica, Beijing University of Chinese Medicine, Beijing, China.

**Keywords:** randomized controlled trials, Salvianolate injection, systematic review, unstable angina pectoris

## Abstract

**Background:**

To systematically evaluate the clinical efficacy and safety of Salvianolate injection in the treatment of unstable angina pectoris (UAP).

**Methods:**

Using literature databases, we conducted a thorough and systematic retrieval of randomized controlled trials (RCTs) that using Salvianolate injection for treating UAP. The Cochrane Risk of Bias Assessment Tool was used to evaluate the methodological quality of the RCTs, and then the data were extracted and meta-analyzed by RevMan5.2 software.

**Results:**

A total of 22 RCTs with 2050 participants were included. The meta-analysis indicated that the combined use of Salvianolate injection and western medicine (WM) in the treatment of UAP can achieve a superior effect in angina pectoris total effective rate (risk ratio [RR] = 1.22, 95% confidence interval [CI] (1.17, 1.27), Z = 10.15, *P* < 0.00001], and the total effectiveness rate of electrocardiogram [RR = 1.26, 95% CI (1.19,1.34), Z = 7.77, *P* < 0.00001]. In addition, Salvianolate injection can improve the nitroglycerin withdrawal rate and the serum level of NO, decrease high-sensitivity C-reactive protein. Adverse drug reactions (ADRs) or adverse drug events (ADEs) were reported in 6 RCTs involving 15 cases; however, there were no serious ADRs/ADEs.

**Conclusion:**

Based on the systematic review, the combined use of Salvianolate injection and WM in the treatment of UAP can achieve a better effect; however, there was no definitive conclusion about its safety. More the large-sample and multicenter RCTs are needed to support its clinical usage.

## Abstract 2

Supplemental Digital Content is available in the text

## 1 Introduction

Unstable angina pectoris (UAP) is a clinical angina pectoris syndrome, which is between stable angina pectoris and acute myocardial infarction.^[1]^ UAP easily exacerbates into acute myocardial infarction and ischemic death, and it includes the initial onset angina pectoris, aggravated effort type angina pectoris, postinfarction angina pectoris, and variant angina pectoris.^[2]^ As a sort of common cardiovascular disease, UAP has complex lesions and rapid progress. Furthermore, its prognosis is multidirectional.^[3]^ Thus, in recent years, it has become a popular research topic all over the world. Some studies have shown that the pathological basis of UAP is unstable plaque, which mainly related to plaque rupture, thrombosis, collateral circulation conditions, and other factors.^[4–7]^

According to the Traditional Chinese Medicine (TCM) theory, UAP belongs to “chest pain,” “heartache,” mainly caused by the deficiency in both Yang and Qi, Blood stasis.^[8–10]^ Recently, Salvianolate injection combined with western medicine (WM) is widely used in the treatment of UAP, and has better effect in eliminating symptoms, controlling angina pectoris, and improving the electrocardiogram (ECG). Salvianolate injection was listed into second-class new patent Chinese medicine by the Chinese State Food and Drug Administration, which is approved in May 25, 2005.^[11]^

Salvianolate injection is prepared from water-soluble compounds, which were extracted from the Danshen Radix, and the main ingredients, namely purified polyphenols acid salts were over 80%.^[12,13]^ The large number of preclinical pharmacology studies and multicenter clinical trials has proved that Salvianolate injection was safe and effective, especially it was stable in quality, because its main ingredient was relatively simple.^[14]^ Salvianolate injection can promoting blood circulation, remove blood stasis, and is mainly used in the treatment of coronary heart disease and angina pectoris.^[15]^ The research showed that Salvianolate had the significant inhibitory effect to Cu^2+^ and oxidation of endothelial cell-mediated low-density lipoprotein, and a strong antioxidant capacity, suggesting that Salvianolate may inhibit the development of atherosclerosis.^[16]^

Currently, there were 3 meta-analyses about Salvianolate injection for treating UAP,^[17–19]^ but their methodological issues defect the credibility of the articles, such as lacking restriction on the onset of disease, significant heterogeneity among interventions. Therefore, by collecting the existing clinical randomized controlled trials’ (RCTs) data, this study aims to present an objective evaluation on the efficacy and safety of Salvianolate injection for UAP, to serve as a reference for clinical treatment.

## 2 Methods

This study was conducted according to the Cochrane risk of bias tool, including literature search, inclusion criteria, data extraction, quality assessment, and statistical analysis.

### 2.1 Literature search

Two independent authors (DZ and SL) performed a systematic literature search in China National Knowledge Infrastructure Database, Chinese Biomedical Literature Database, Chinese Scientific Journals Database (VIP), Wanfang Database, PubMed, and the Cochrane Library without any restrictions to languages and calendar date. In the Chinese databases, the terms “ 
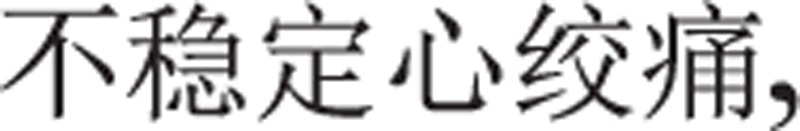
” “ 
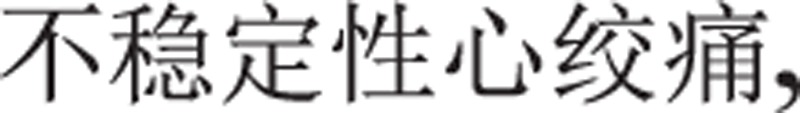
” and “ 
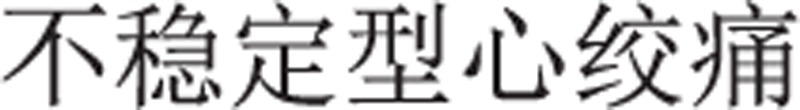
” were used as subject terms for the initial search, and “ 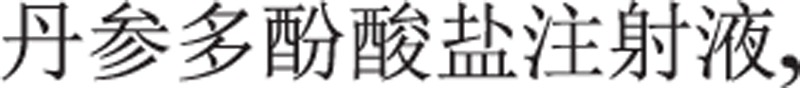
” “ 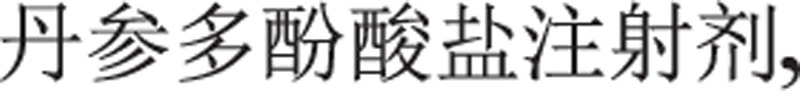
” and “ 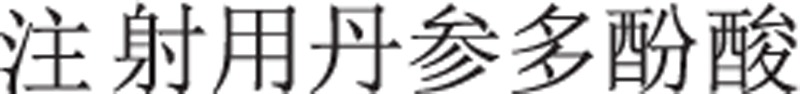
” were used to search again among above results. In English databases, the Mesh terms of “Salvianolate injection” were used as subject words for the initial search, and “unstable angina pectoris” were used for further retrieval. The search strategy in PubMed was below.

(1) “Anginas, Unstable” [Mesh]
(2) “Unstable Anginas” [Title/Abstract] OR “Unstable Angina Pectori∗” [Title/Abstract] OR “Unstable Angina” [Title/Abstract] OR “Angina at Rest” [Title/Abstract] OR “Preinfarction Angina∗” [Title/Abstract] OR “Myocardial Preinfarction Syndrome∗” [Title/Abstract]
(3) (1) OR (2)
(4) “Salvianolate injection” [Title/Abstract] OR “Dan Shen Duo Fen Suan Yan” [Title/Abstract]
(5) (3) AND (4)

### 2.2 Inclusion criteria

Only RCTs meeting the following criteria were included in the systematic review: Clinical RCTs regarding Salvianolate injection for treating UAP regardless of blinding. Participants included in the study met the diagnostic criteria for “diagnosis and treatment of unstable angina pectoris” released by Chinese Society of Cardiovascular Diseases of Chinese Medical Association in 2000 and 2007,^[20,21]^ and “the ischemia heart disease naming and diagnosis standard” released by World Health Organization in 1979.^[22]^ All the patients had recurrent angina pectoris records and changes of ECG ischemic ST-T, and no limitations placed on gender, race, age, course of disease, and severity of disease. In the control group, the commonly used WM were β-receptor antagonists, nitric acid ester medicines, calcium antagonists, aspirin, and so on. The experimental group was treated by WM on the same basis with the control group, but combining with Salvianolate injection. Patients with complications were given corresponding treatment. There was no other Chinese medicine, acupuncture, or surgery performed in any groups. The main interventions were Salvianolate injection + WM versus WM. The criterion of therapeutical effect met “clinical research guideline of medicine for cardiovascular system” released by Clinical pharmacology base of cardiovascular system in the Ministry of Health in 1988.^[23]^ The primary outcomes were angina pectoris total effective rate and the total effectiveness rate of ECG, which calculated by the following formula: the total effective rate = (number of significantly effective patients + number of effective patients)/total number of patients × 100%. For the angina pectoris total effective rate, the patient was regarded as significantly effective when attack frequency of angina pectoris reduced more than 80%, nitroglycerin consumption reduced more than 80%. The patient was regarded as effective when attack frequency of angina pectoris and nitroglycerin consumption reduced by 50% to 80%. The patient was regarded as invalid when attack frequency of angina pectoris and nitroglycerin consumption were <50%, or attack frequency, degree, and the duration of angina pectoris were aggravating, nitroglycerin consumption increased. As for the total effectiveness rate of ECG, the patient was regarded as significantly effective when original low ST segment or T wave inversion of resting ECG returned to normal. The patient was regarded as effective when the ST segment was recovered in 0.05 to 0.10 mV, but did not reach the normal, or inverted T wave became shallow more than 50% or T wave became upright from flat after treatment. The patient was regarded as invalid when resting ECG was basically the same or not met the above indicators no matter before and after the treatment, or ST segment and inverted T wave became worse than original ones. Secondary outcomes were the nitroglycerin withdrawal rate (NWR), and serum level of NO, high-sensitivity C-reactive protein (hs-CRP), and adverse drug reactions (ADRs)/adverse drug events (ADEs).

### 2.3 Data extraction and quality assessment

Two researchers (DZ and JW) independently read the titles and abstracts of the identified RCTs, excluding not relevant ones, reviews, and pharmacological experiments. For controlled trials, the full text was read before deciding whether it should be included. The 2 researchers conducted their quality evaluations independently; any disagreement was resolved by a third researcher (SL). The quality assessment was conducted by the Cochrane Risk of Bias Assessment Tool, which included random sequence generation, allocation concealment, blinding of participants and personnel, blinding of outcome assessment, incomplete outcome data, selective outcome reporting, and other sources of bias.^[24]^ Each RCT was rated “high,” “unclear,” or “low.” “High” referred to incorrect random methods, no allocation concealment or no blinding. “Unclear” referred to no description in the text with which to assess bias. “Low” referred to correct random methods, appropriate blinding without being violated through implementation, and detailed description in the RCT.

Finally, ethical approval was not necessary in current meta-analysis because our meta-analysis just gathered the RCTs from literature search, this procedure was without deal with any patients’ personal data and harm to any patient.

### 2.4 Statistical analysis

RevMan5.2 software^[25]^ was used for the data analysis, with risk ratio (RR) as a dichotomous indicator, mean difference (MD) to measure continuous variables, 95% confidence intervals (95% CIs) to present the data, chi-squared to analyze heterogeneity among the various studies, and I^2^ to evaluate the degree of heterogeneity. When *P* > 0.1 and *I*^2^ < 50%,^[26]^ a meta-analysis was conducted using the fixed-effect model; otherwise, the random-effect model was used. And the difference was statistically significant between groups on the condition of *P* < 0.05. When clinical or methodological heterogeneity existed, such as the differences of dosage, course, and so on, subgroup analysis was used. If the number of included RCTs was available, sensitivity analysis was performed to test the stability of results, and a funnel chart was chosen to analyze potential publication bias.

## 3 Results

### 3.1 Analysis of literature and assessment of quality

**Figure 1:**
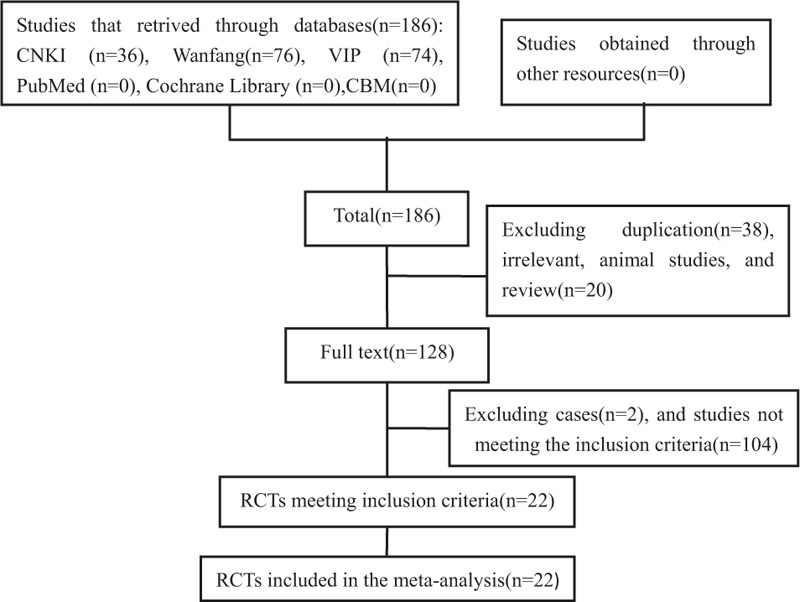
Flow chart of literature search.

During the initial selection, 214 articles were retrieved. By reading titles and abstracts, duplications, apparently unrelated articles, nonclinical studies, reviews, and commentaries were excluded. A total of 128 articles about clinical study on Salvianolate injection's efficacy for treating UAP were identified. After reading the full text, by removing case studies and articles that did not meet the inclusion criteria, 22 RCTs were included. All the included RCTs were in Chinese (Fig. 1). The 22 RCTs included 2050 cases, among which 1035 cases were in the experimental groups, while 1015 in the control groups. The age of patients ranged from 37 to 94 years old, and the average age was 61.10 years old. In the included RCTs, the Salvianolate injection was mainly produced by Shanghai Greenvalley pharmaceutical manufacturers. The majority of its dosage was 200 mg/kg d.^[27–38,41–47]^ The course was almost within 2 weeks. More details regarding the individual trials were presented in Table 1.

**Table 1:**
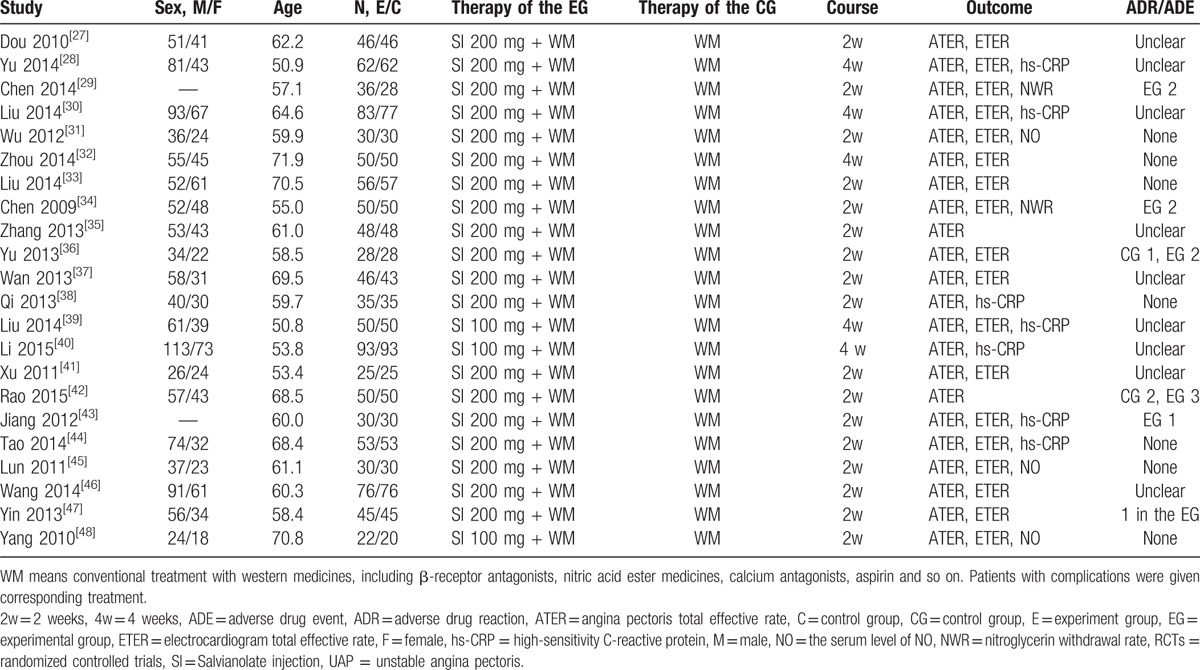
Characteristics of 22 included RCTs on Salvianolate injection for UAP.

**Figure 2:**
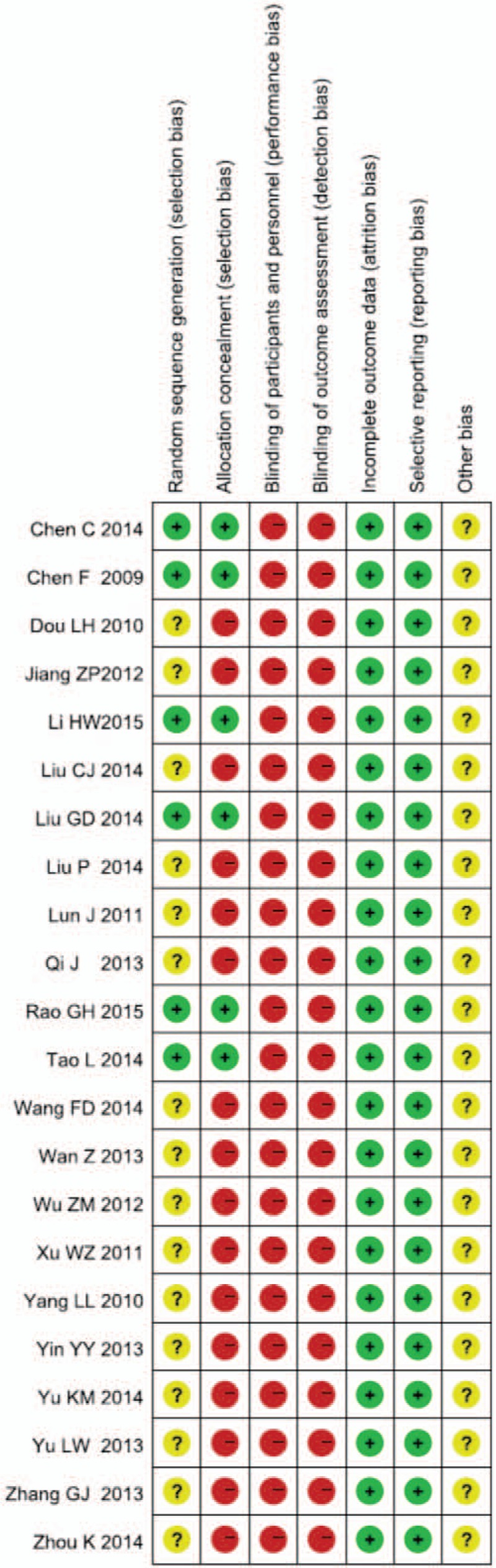
Risk of bias summary.

We used the Cochrane Risk of Bias Assessment Tool to conduct a quality evaluation on the included RCTs, and the results showed that 6 RCTs described the method to generate the allocation sequence, among which 5 RCTs used the random figure table and 1 RCT used the computer random grouping method.^[29,30,34,40,42,44]^ The remaining RCTs were only referred to randomly divided into 2 groups. All of RCTs did not provide information on blinding. None of the included RCTs selected studies to presented reports on incomplete or missing data. In a summary, the overall quality of the included RCTs was generally not high. Details on risk of bias were shown in Fig. 2, Table S1.

### 3.2 Outcomes

All of the RCTs were divided into subgroups caused by the differences in the course. The results of each subgroup were analyzed as follows.

#### 3.2.1 Angina pectoris total effective rate

Two weeks (2w) subgroup: there were 17 RCTs in the subgroup.^[27,29,31,33–38,41–48]^ The test for heterogeneity (*P* = 0.88 > 0.1, *I*^2^ = 0% < 50%) indicated small heterogeneity among the RCTs. Therefore, the fixed model was chosen. The results showed that compared with the control group, the combining use of Salvianolate injection and WM for treating UAP can significantly improve the angina pectoris total effective rate, and statistically significant difference was observed between groups (RR = 1.22, 95% CI [1.17, 1.28], Z = 8.49, *P* < 0.00001).

Four weeks (4w) subgroup: there were 5 RCTs in the subgroup.^[28,30,32,39,40]^ The test for heterogeneity (*P* = 0.35 > 0.1, *I*^2^ = 0% < 50%) found small heterogeneity among the RCTs. Therefore, the fixed model was chosen. The results showed that compared with the control group, the combining use of Salvianolate injection and WM for treating UAP can significantly improve the angina pectoris total effective rate. The statistical difference was significant between 2 groups (RR = 1.21, 95% CI [1.13, 1.30], Z = 5.56, *P* < 0.00001).

**Figure 3:**
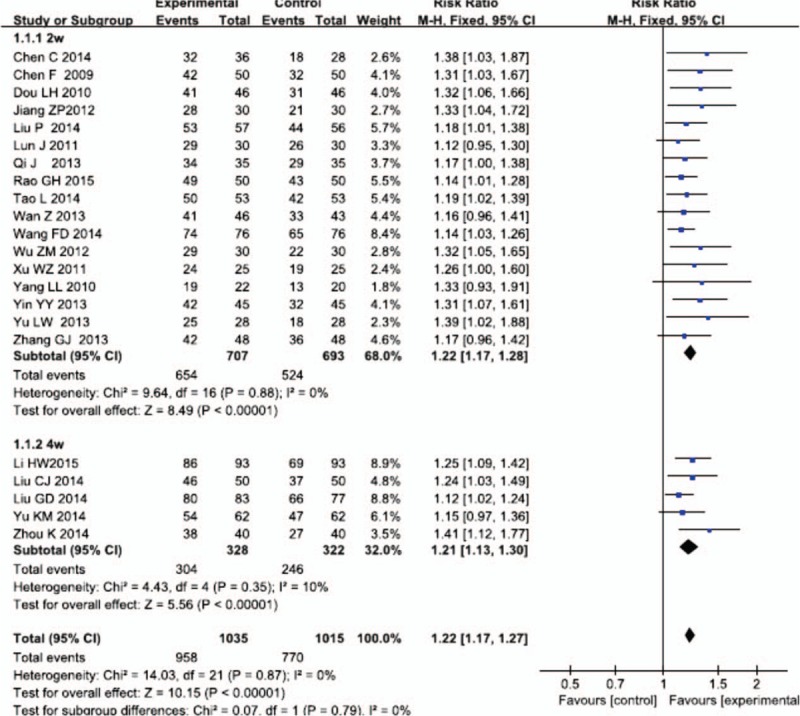
Meta-analysis for comparison of angina pectoris total effective rate between Salvianolate injection + WM and WM. WM = western medicine.

The result of test for heterogeneity between the 2 subgroups was *P* = 0.79 > 0.1, *I*^2^ = 0% < 50%. Accordingly, we could merged the results showed that combining using Salvianolate injection and WM was more effective than single using WM in the treatment of UAP. There was statistically significant difference between 2 groups (RR = 1.22, 95% CI [1.17, 1.27], Z = 10.15, *P* < 0.00001, Fig. 3).

#### 3.2.2 Sensitivity analysis

Sensitivity analysis of angina pectoris total effective rate was conducted, which has done by excluding RCTs with the most and least weighted, or changing analysis effects model to reanalyze the data. After removing the most weighted RCT,^[40]^ the result was RR = 1.22, 95% CI (1.17, 1.27), *P* < 0.00001, and the result of removing the least weighted RCT^[48]^ was RR = 1.22, 95% CI (1.17, 1.27), *P* < 0.00001. The result of changing the mode was RR = 1.20, 95% CI (1.15, 1.24), *P* < 0.00001, which indicated that there was no clinically or statistically qualitative changes. Therefore, it can be concluded that the sensitivity was not high and the results were relatively stable.

#### 3.2.3 Reporting bias

**Figure 4:**
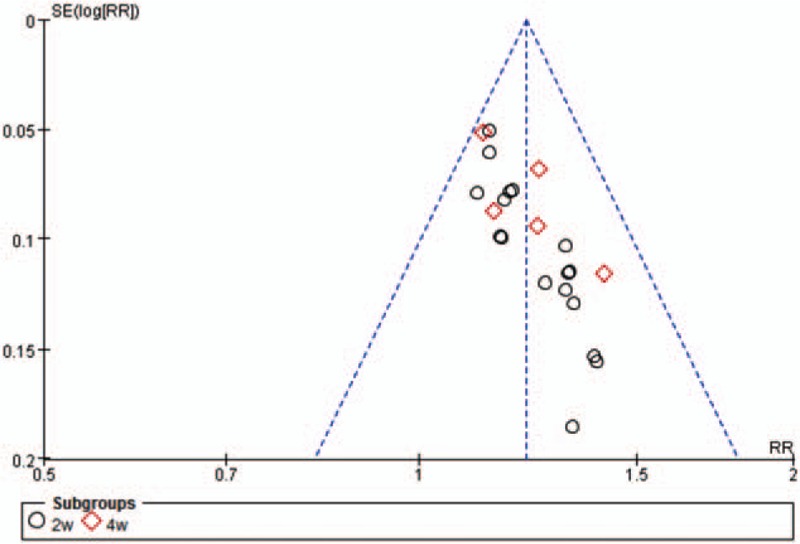
Funnel plot of 2w and 4w subgroups. 2w = 2 weeks, 4w = 4 weeks.

Funnel chart of 2w and 4w subgroups, angina pectoris total effective rate analysis showed potential publication bias of included trials (Fig. 4).

#### 3.2.4 Total effectiveness rate of ECG

Two weeks subgroup: There were 13 RCTs in the subgroup.^[27,29,31,33,34,36,37,41,43–47]^ The test for heterogeneity (*P* = 0.70 > 0.1, *I*^2^ = 0% < 50%) indicated none heterogeneity among the RCTs. Therefore, the fixed model was chosen. The results showed that compared with the control group, the combining use of Salvianolate injection and WM for treating UAP can significantly improve the total effectiveness rate of ECG, and statistically significant differences were observed between groups (RR = 1.28, 95% CI [1.19, 1.37], Z = 7.11, *P* < 0.00001).

Four weeks subgroup: There were 3 RCTs in the subgroup.^[30,32,39]^ The test for heterogeneity (*P* = 0.18 > 0.1, *I*^2^ = 41% < 50%) indicated small heterogeneity among the RCTs. Therefore, the fixed model was chosen. The results showed that compared with the control group, the combining use of Salvianolate injection and WM for treating UAP can significantly improve the total effectiveness rate of ECG, and statistically significant differences were observed between groups (RR = 1.21, 95% CI [1.10, 1.34], Z = 3.72, *P* = 0.0002).

**Figure 5:**
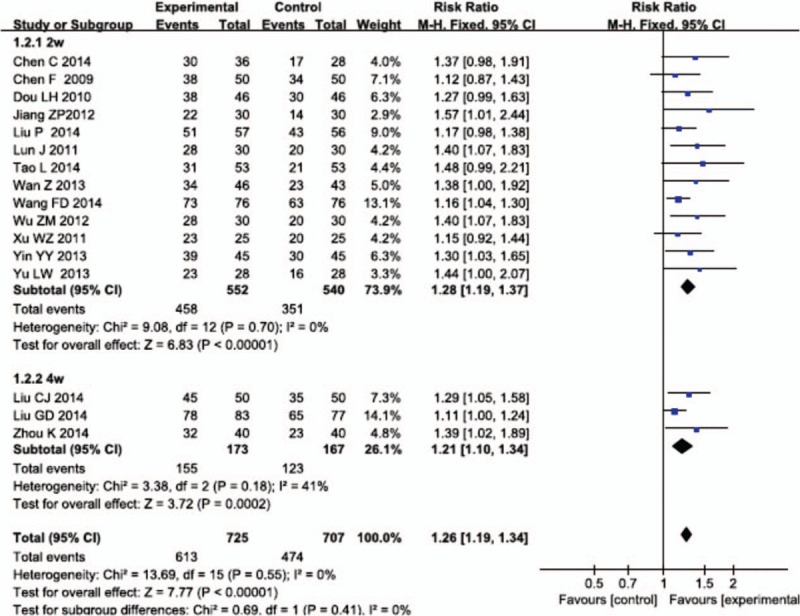
Meta-analysis for the total effectiveness rate of electrocardiogram between Salvianolate injection + WM and WM. WM = western medicine.

The result of test for heterogeneity between the 2 subgroups was *P* = 0.41 > 0.1, *I*^2^ = 0% < 50%. Accordingly, we could merged the results showed that combining using Salvianolate injection and WM was more effective than single using WM in the treatment of UAP. There was statistically significant differences between 2 groups (RR = 1.26, 95% CI [1.19, 1.34], Z = 7.77, *P* < 0.00001) (Fig. 5).

#### 3.2.5 Serum level of hs-CRP

Two weeks subgroup: There were 3 RCTs in the subgroup.^[38,43,44]^ The test for heterogeneity (*P* = 0.08 < 0.1, *I*^2^ = 61% > 50%) indicated some heterogeneity among the RCTs, so the random model was used. The results showed that compared with the control group, the combining use of Salvianolate injection and WM for treating UAP can significantly reduce the serum level of hs-CRP, and statistically significant differences were observed between groups (MD = −1.53, 95% CI [−1.90, −1.16], Z = 8.05, *P* < 0.00001).

Four weeks subgroup: There were 4 RCTs in the subgroup.^[28,30,39,40]^ The test for heterogeneity (*P* < 0.00001, *I*^2^ = 99% > 50%) found obvious heterogeneity among the RCTs, so the random model was used. The results showed that compared with the control group, the combining use of Salvianolate injection and WM for treating UAP can significantly reduce the serum level of hs-CRP, and the difference between the 2 groups was significant (MD = −1.74, 95% CI [−2.88, −0.60], Z = 3.00, *P* = 0.003).

**Figure 6:**
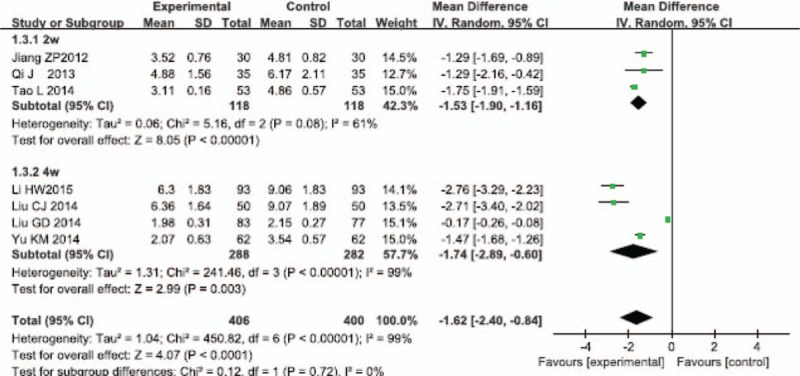
Meta-analysis for comparison of the serum level of high-sensitivity C-reactive protein between Salvianolate injection + WM and WM. WM = western medicine.

After all, we merged the results of subgroups because there was no obvious heterogeneity between the 2 subgroups (*P* = 0.72 > 0.1, *I*^2^ = 0% < 50%). The difference between the experimental group and the control group was significant (MD = −1.62, 95% CI [−2.39, −0.84], Z = 4.07, *P* < 0.00001) (Fig. 6).

#### 3.2.6 Other outcomes

There were 2 RCTs mentioned NWR.^[29,34]^ The test for heterogeneity (*P* = 1.00 > 0.1, *I*^2^ = 0% < 50%) found none heterogeneity among the RCTs, so the fixed model was chosen. The results showed that the experimental group was more effective than the control group and the difference between the 2 groups was significant (MD = 1.22, 95% CI [1.00, 1.48], Z = 1.99, *P* < 0.05). And the 2 RCTs also mentioned the serum level of NO.^[29,44,46]^ The test for heterogeneity (*P* < 0.00001, *I*^2^ = 99% > 50%) found obvious heterogeneity among the RCTs, so the random model was chosen. The results showed that the experimental group was more effective than the control group, and the difference between the 2 groups was significant (MD = 22.49, 95% CI [3.11, 41.87], Z = 2.27, *P* = 0.02<0.05).

#### 3.2.7 Safety

Among the included RCTs, 7 RCTs indicated that there were no ADRs/ADEs.^[31–33,38,44,45,48]^ Six RCTs reported 15 ADRs/ADEs, among which 11 cases were from the experimental groups, while 4 from the control groups.^[29,34,36,42,43,47]^ ADRs/ADEs included headache, dizziness, gastrointestinal reaction, and so on. There was no significant difference about ADRs/ADEs between the 2 subgroups. However, none of severe ADRs occurred. The remaining 9 RCTs did not mention ADRs/ADEs. Accordingly, we could not conclude that Salvianolate injection was absolutely safe.

## 4 Discussion

After analysis of the RCTs regarding Salvianolate injection in the treatment of UAP, we demonstrated that Salvianolate injection was effective in the treatment of UAP. First, compared with using WM only, the combined use of Salvianolate injection and WM can improve the angina pectoris total effective rate and the total effectiveness rate of ECG. Second, the combined use of Salvianolate injection and WM therapy can improve the NWR and the serum level of NO, decrease hs-CRP. And there were statistically significant differences between the experimental group and the control group. From the results of 2w and 4w subgroups’ analysis, neither angina pectoris total effective rate nor the total effectiveness rate of ECG of 2 subgroups showed statistically significant difference. In terms of ADRs/ADEs, there was no definite conclusion about safety for 4w clinical treatment, because that 4 RCTs of 4w subgroup had unclear ADRs/ADEs records. In a summary, the results of subgroup analysis showed that long duration neither significantly improve the efficacy nor reduce the risk about safety. Furthermore, the drug instructions of Salvianolate injection recommended the course is 2w. Thus, our study suggested that the course of Salvianolate injection should be in strict accordance with the drug instructions. Moreover, modern pharmacological research findings confirmed that major pharmacological effects of Danshen Radix include antiatherosclerosis, reducing myocardial ischemia, and infarct size.^[48]^ And the result of this meta-analysis was supported by the findings of previous research.^[17–19]^ Therefore, our study suggested that Salvianolate injection can be widely used in clinics.

There were 3 related systematic reviews in database.^[17–19]^ Two of them were published in 2013. In Guan Yan's study, the control group was also used other injection on the basis of conventional medicine, such as Danshen injection and Danhong injection.^[17]^ In our inclusion criteria, the experimental group only used Salvianolate injection combined with WM, the control group used WM single, just for avoiding other TCM preparations possible interference on the results. And the RCTs’ quality assessment was conducted by the Cochrane Risk of Bias Assessment Tool. Our study not only updated the latest RCTs but also analyzed the differences of 2w and 4w courses.

Salvianolate injection had definite effects on the treatment of UAP; however, the medical staff should be cautious on its clinical use to avoid and reduce serious ADRs/ADEs. Among included RCTs, our study reported 15 ADRs, 11 cases were from the experimental groups, while 4 from the control groups. ADRs included headache, dizziness, gastrointestinal reaction, and so on. Some research showed that TCM injections were in high proportion of Chinese medicine about ADRs. Some TCM injection combined with solvent was easily occur incompatibility, even insoluble particles significantly higher than the blank solvent.^[49]^ The studies of Wang and Ren showed that Salvianolate injection should not be compatible with a variety of injection, such as vitamin C injection, Astragalus injection, Isosorbide Mononitrate injection, and so on.^[50,51]^ According to foreign research, it was common in clinical use that the applications of drugs often beyond the instructions including extended indication, increased dosage, extended course.^[52,53]^ Therefore, based on the complexity of the TCM injection component, it is recommended that the clinical usage of Salvianolate injection should be in strict accordance with the instructions, in order to avoid the occurrence of compatibility and ADRs.

## 5 Limitations

This study has several limitations. First, the review included a total of 22 RCTs; however, the overall quality of the included RCTs was generally not high, and they lacked of large-scale RCTs. The majority of the studies marked “random” only without describing specific random allocation method and blinding. Therefore, the evaluation of Salvianolate injection's efficacy and safety in treating UAP still requires higher-quality clinical studies and further discussions. We should pay close attention to the rationality of the clinical application and safety of Salvianolate injection, and attach great importance to the scheme design, random allocation, blinding method of such research and provide more evidence for clinical application. Second, the systematic review included only published studies in the database, with no the relevant gray literature, which possibly cause a selection bias in the literature. And the included RCTs were performed in patients of Asian descent; therefore, it is unclear whether the conclusions of our study apply to other populations. Third, the 22 RCTs did not report the long-term efficacy and follow-up. This might lead to an inadequate assessment to the clinical efficacy. Generally speaking, our findings should be confirmed by large-sample and multicenter RCTs or real-world studies.

## 6 Conclusions

In conclusion, our results showed that the combining use of Salvianolate injection and WM had superior efficacy in treating UAP. However, since the RCTs enrolled in the study were not of high quality, more larger-sample and multicenter RCTs should be conducted to consolidate our findings.

## Supplementary Material

Supplemental Digital Content

## References

[1] Hui JK Advances in treatment of unstable angina. Chin J Ger Care 2009;2:69–71.

[2] Wang YL Xu DJ Description of the mechanism and treatment about unstable angina pectoris. Guide China Med 2010;32:33–4.

[3] Luo J Research progress of unstable angina. Adv Cardiovasc Dis 2000;5:301–5.

[4] Chinzchiss Current therapeutic strategies in unstable angina. Eur Heart J 1999;1:12–6.

[5] Kang CL Yang JG Research progress of unstable angina. J Clin Cardiol 1999;9:19–23.

[6] Han YJ Sheng XG Current treatment of unstable angina pectoris. J Trad Chin Med 2007;10:945–8.

[7] Ni KS Clinical progress of unstable angina. Adv Cardiovasc Dis 1994;2:78–9.

[8] Guo JS Integrative treatment of unstable angina. Mod J Integr Trad Chin West Med 2008;25:3933–4.

[9] Huang YS Teaching and Clinical in Traditional Chinese Internal Medicine. Vol. 67. 1999;Beijing:People's Health Publishing House, 27–29.

[10] Ji Q Luo YQ Wang WH Research advances in Traditional Chinese Medicine syndromes in cancer patients. J Integr Med 2016;1:12–21. 10.1016/S2095-4964(16)60237-6 26778224

[11] Yang ZX Lin Q Ma L Salvia literature research on the pathological effects of cardiovascular disease. World J Integr Trad West Med 2012;2:93–7.

[12] Kashima Tanaka M Tsujimoto Y Kawamoto K Generation of free radicals and/or active oxygen by light or irradiation of hydrogen peroxide or sodium hypochlorite. J Endod 2003;2:141–3. 10.1097/00004770-200302000-00013 12597716

[13] Xu J Fan WH Effect of Salvianolate on migration of human vascular endothelial cells. J China Integr Med 2003;3:211–3. 10.3736/jcim20030320 15339565

[14] Miao Y Gao ZH Xu FQ Clinical observation on Salvianolate for the treatment of angina pectoris in coronary heart disease with heart-blood stagnation syndrome. Trad Chin Drug Res Clin Pharm 2006;2:140–4.

[15] Peng L Salvia polyphenol salt coagulated with decompensated cirrhosis. Guide J Trad Chin Med Pharm 2009;6:22–4.

[16] Fang XL Pharmacology and clinical application of salvia polyphenol salt. Chin Med 2012;10:1343–4.

[17] Guan Y Liu B Liao XJ Efficacy and safety of Salvianolate injection in the treatment of unstable angina pectoris. Guide China Med 2013;19:50–2.

[18] Wu HB Meta-analysis of Salvianolate injection in the treatment of unstable angina pectoris. Mod Pract Med 2013;2:187–9.

[19] Yan GP Zhu CL Sun YQ Meta-analysis of Salvianolate injection in the treatment of unstable angina pectoris. J Emerg Trad Chin Med 2015;5:771–4.

[20] Chinese Society of Cardiovascular Diseases of Chinese Medical Association, Editorial Committee of Chinese Journal of Cardiology Diagnosis and treatment of unstable angina pectoris. Chin J Cardiol 2000;6:8–11.

[21] Chinese Society of Cardiovascular Diseases of Chinese Medical Association, Editorial Committee of Chinese Journal of Cardiology Unstable angina and non-ST segment elevation myocardial infarction diagnosis and treatment guidelines. Chin J Cardiol 2007;4:295–304.

[22] International Heart Disease Academic Society, The Association and the World Health Organization (ISFC/WHO), The Clinical Naming Standardization Union Group Formulates The ischemia heart disease naming and diagnosis standard. Circulation 1979;59:607–8.761341

[23] Editorial Committee of Chinese Journal of Cardiology Clinical research guideline of medicine for cardiovascular system. Chin J Clin Pharm 1988;4:245–55.

[24] Julian PTH Douglas GA Peter C The Cochrane Collaboration's tool for assessing risk of bias in randomized trials. BMJ 2011;343:d5928–9. 22008217 10.1136/bmj.d5928 PMC3196245

[25] The Cochrane Collaboration Review Manager (RevMan) (Computer Program). Version 5.2. Copenhagen:The Nordic Cochrane Centre, The Cochrane Collaboration; 2012.

[26] Zheng MH Applications and Example Analyzation of the Meta-Analysis Software. 2013;Beijing:People's Medical Publishing House, 4–5.

[27] Dou LH Salvianolate injection in the treatment of unstable angina pectoris. Chin J Exper Trad Med Form 2010;16:206–7.

[28] Yu MK Jiao LQ Effect of Salvianolate injection on serum levels of C reactive protein, tumor necrosis factor and soluble cell apoptosis in patients with unstable angina pectoris. Hebei J Trad Chin Med 2014;1:15–6.

[29] Chan C Dai HY Yin LZ Clinical study on the treatment of unstable angina pectoris with Salvianolate injection. China Foreign Med Res 2014;31:5–7.

[30] Liu GD Xiao GL Effect of Salvianolate injection on C-reactive protein and blood rheology in patients with unstable angina pectoris. J Emerg Trad Chin Med 2014;1:3–5.

[31] Wu ZM Luo XY Wang SH The effect of Salvianolate injection on NO, ET-1 and CRP in the treatment of unstable angina pectoris. Henan Trad Chin Med 2012;7:864–5.

[32] Zou K Wang M Effect of Salvianolate injection on serum VEGF and MMP-9 in aged patients with coronary heart disease. Chin J Clin Ratio Drug Use 2015;1:21–3.

[33] Liu P Observation on the therapeutic effect of combined with western medicine and Salvianolate injection in treating unstable angina pectoris. Guide J Trad Chin Med Pharm 2014;11:72–3.

[34] Chen F Ni L Xu P Salvianolate injection in the treatment of 50 cases unstable angina pectoris. Pract J Card Cereb Pneum Vascr Dis 2009;11:9589–90.

[35] Zhang GJ Salvianolate injection in the treatment of 96 cases unstable angina pectoris. China Foreign Med Treat 2013;18:118–22.

[36] Yu LW Effect of Salvianolate injection in the treatment of unstable angina. Pract Clin Med 2013;7:23–6.

[37] Wang Z Tong M Salvianolate injection in the treatment of elderly patients with unstable angina clinical research. Pract Clin J Integr Trad Chin West Med 2013;4:11–3.

[38] Qi J Guo ZH Salvianolate injection in the treatment of 35 cases unstable angina pectoris. Hunan J Trad Chin Med 2013;5:23–6.

[39] Liu CJ Li HW Ning W Salvianolate injection in the treatment of unstable angina pectoris and effect on serum lipids and inflammatory factors. Mod J Integr Trad Chin West Med 2014;13:1394–6.

[40] Li HW Liu CJ Zhang YQ Efficacy and nursing care of Salvianolate injection in the treatment of unstable angina pectoris. Chin J Hypertens 2015;1:454–5.

[41] Xu WZ Xu KL Fu YD Efficacy of Salvianolate injection in the treatment of heart-blood stagnation angina pectoris. China Health Ind 2011;8:7–8.

[42] Rao GH Xi LW Observation on the effect of treating coronary heart disease angina pectoris with Salvianolate injection. Strait Pharm J 2015;5:136–8.

[43] Jiang ZP Ji XW Zhang AY Observation on the effect of treating coronary heart disease angina pectoris with Salvianolate injection. J Clin S (Electron Ed) 2012;19:6097–8.

[44] Tao L Song CY Chen D Effect of Salvianolate injection on the treatment of unstable angina pectoris and its effect on serum C reactive protein. Chin J Clin Ratio Drug Use 2014;6:46–7.

[45] Lun J Ji XW Zhang AY Effect of Salvianolate injection in the treatment of unstable angina pectoris and effect on serum C-reactive protein. Chin J Clin Ratio Drug Use 2011;2:1–2.

[46] Wang FD Effect observation of Salvianolate injection in the treatment of unstable angina pectoris and the effect of the inflammatory factors. Strait Pharm J 2014;6:104–5.

[47] Yin YY Xu HB Observation on the clinical effect of treating unstable angina pectoris with Salvianolate injection. J Changzhi Med Coll 2013;3:188–91.

[48] Zhang C Zhao ZY Functional mechanism research of *Salvia miltiorrhiza* and prescription treating the coronary heart disease based on the TCM syndrome differentiation. Chin J Basic Med Trad Chin Med 2014;4:462–4.

[49] Chinese Pharmacopoeia 2005. Volume 2: Appendix C.

[50] Chinese Pharmacopoeia Commission Chinese Pharmacopoeia. Vol 4. 2015;Beijing:China Medical Science Press, 4–5.

[51] Ren X Xie N Xu XY Study on compatibility and stability of 21 kinds of clinical common drugs used for Salvianolate injection. China Pharm 2012;2:22–5.

[52] Huang L Shen XL Chen L Correct understanding and effectively regulate the behavior of ultra-medication instructions. Chin J Hosp Pharm 2009;11:949–51.

[53] Gazarian M Kelly M McPhee JR Off-label use of medicines: consensus recommendations for evaluating appropriateness. Med J Aust 2006;10:544–8. 10.5694/j.1326-5377.2006.tb00689.x 17115966

