# Supplementary material for: Salvianolate injection in the treatment of unstable angina pectoris: A systematic review and meta-analysis
Source: Medicine (Baltimore). 2016 Dec 23;95(51):e5692. doi: 10.1097/MD.0000000000005692 (PMC5181825; doi:10.1097/MD.0000000000005692)
Supplement: Supplemental Digital Content [file medi-95-e5692-s001.doc]

Table. The methodological qualities of all the included studies

| Study ID | Sequence generation | Allocation concealment | Blinding of participants and  personnel | Blinding of outcome  assessment | Incomplete outcome data | Selective outcome reporting | Other sources of bias |
| --- | --- | --- | --- | --- | --- | --- | --- |
| Dou 2010^27^ | Unclear | High | High | High | Low | Low | Unclear |
| Yu 2014^28^ | Unclear | High | High | High | Low | Low | Unclear |
| Chen2014^29^ | Low | Low | High | High | Low | Low | Unclear |
| Liu 2014^30^ | Low | Low | High | High | Low | Low | Unclear |
| Wu 2012^31^ | Unclear | High | High | High | Low | Low | Unclear |
| Zhou2014^32^ | Unclear | High | High | High | Low | Low | Unclear |
| Liu 2014^33^ | Unclear | High | High | High | Low | Low | Unclear |
| Chen2009^34^ | Low | Low | High | High | Low | Low | Unclear |
| Zhang2013^35^ | Unclear | High | High | High | Low | Low | Unclear |
| Yu 2013^36^ | Unclear | High | High | High | Low | Low | Unclear |
| Wan 2013^37^ | Unclear | High | High | High | Low | Low | Unclear |
| Qi 2013^38^ | Unclear | High | High | High | Low | Low | Unclear |
| Liu 2014^39^ | Unclear | High | High | High | Low | Low | Unclear |
| Li 2015^40^ | Low | Low | High | High | Low | Low | Unclear |
| Xu 2011^41^ | Unclear | High | High | High | Low | Low | Unclear |
| Rao 2015^42^ | Low | Low | High | High | Low | Low | Unclear |
| Jiang2012^43^ | Unclear | High | High | High | Low | Low | Unclear |
| Tao 2014^44^ | Low | Low | High | High | Low | Low | Unclear |
| Lun 2011^45^ | Unclear | High | High | High | Low | Low | Unclear |
| Wang2014^46^ | Unclear | High | High | High | Low | Low | Unclear |
| Yin 2013^47^ | Unclear | High | High | High | Low | Low | Unclear |
| Yang2010^48^ | Unclear | High | High | High | Low | Low | Unclear |
